# Clear Waters, Bright Futures: Do Low‐Cost Information Interventions Increase Health Preventive Behaviors

**DOI:** 10.1002/hec.4977

**Published:** 2025-05-20

**Authors:** Rafi Amir‐ud‐Din, Muhammad Khan, Zahra Murad, Irene Mussio

**Affiliations:** ^1^ Department of Economics COMSATS University Islamabad Lahore Pakistan; ^2^ Department of Accounting, Economics and Finance University of Portsmouth Portsmouth UK; ^3^ UNEC Cognitive Economics Centre Azerbaijan State University of Economics Baku Azerbaijan; ^4^ Department of Economics Leeds University Business School Leeds UK

**Keywords:** behavioral change, health, information provision, RCT, water contamination

## Abstract

Contaminated drinking water poses a significant, long‐term health challenge in developing countries. With the aim of shedding light on the most effective presentation of this information in awareness campaigns, we run a randomized control trial involving 1388 households in Punjab, Pakistan. We provide information about fecal matter (E.Coli) presence in drinking water and on ways to treat water to make it potable. This intervention increases the likelihood of adopting in‐home water purification for those households who were provided with information about water contamination results. Those informed of both water contamination and potential water treatment methods exhibit an even higher likelihood of behavior change. This study is evidence of the potential efficacy of low‐cost information‐based interventions, offering valuable insights for health policy in resource‐constrained settings.

## 1 Introduction

Around 2 billion people in the world use contaminated drinking water (Bain et al. 2020), which causes diseases like diarrhea, dysentery, cholera or typhoid fever. The World Health Organization estimates that around 829,000 people die each year from diarrhea as a result of unsafe drinking‐water, poor sanitation and hand hygiene (World Health Organization 2014, 2022; Liu and Yang 2012). Contaminated water is also one of the top causes of death for children under five (Baker et al. 2016; Bertuzzo and Mari 2017; Haushofer et al. 2021) and impacts maternal health (Danagoulian and Jenkins 2021). Consistent adoption of safe and clean drinking water is vital for public health and it is explicitly recognized in the United Nations Sustainable Development Goal (SDG) 6, aiming to achieve universal and equitable access to safe and affordable drinking water for all (United Nations in Pakistan 2024). It can also improve gender equality (SDG 5), strengthening the link between the SDGs (Dickin et al. 2021).

In contrast to traditional water‐sanitation approaches, our study in Pakistan introduces a new angle by relying on information provision. While past trials have provided participants with items such as water treatment solution (Dupas et al. 2023) or water filters (Brown et al. 2008; T. F. Clasen et al. 2004; Fagerli et al. 2020), we are investigating whether simply sharing information can motivate households to take action on water safety as a preventative action (Bennett et al. 2015; Whittington et al. 2012). Rooted in the concept of preventative health, our study not only reframes the narrative on water sanitation but also presents a low‐cost approach to enhancing public health, while fostering both wellbeing and equality (Günther and Schipper 2013; Gimelli et al. 2018; Mkupete et al. 2022).

In the case of Pakistan, despite 92% of the country's population being covered by a water supply system, only 36% of the water supply is considered safe for consumption (UNICEF 2023). The quality of this service is poor, intermittent, and supply networks are typically plunged into sewers (Khan et al. 2013; Nabeela et al. 2014), and government measures to improve the water quality are unlikely to happen in the short‐term, given the level of investment needed. Moreover, sources of water in low‐income and rural areas are more likely to be contaminated either with fecal matter or with agricultural pollutants (Ogunyoku et al. 2011; Lai 2017). Thus, it is necessary to find low‐cost ways of safe drinking water that happen through household investment in water treatment. A prior study showed that supplying solar water disinfection facilities to 600 households in the districts of Faisalabad and Hyderabad reduced the incidence of diarrhea in children under 5 years of age by 40.1% and 19.4% (Nabeela et al. 2014). Our study focuses on how targeted information in the form of water test results affects households' preventative health behaviors with respect to water purification. Specifically, households in 24 villages in Pakistan were randomly assigned to three information provision treatment arms. Those who were provided the partial information treatment were only given the results of the test for water quality (whether fecal contamination was found or not). Those in the full information treatment were additionally provided with the information on water purification options at home (i.e. point of use purification).

The disinfection of water at the point of use is a solution for water quality increase which is endorsed by the World Health Organization (Shaheed et al. 2018). For areas where drinking water is contaminated, investing in water purification is a sustained effort to treat water at the point of use which could have positive and long‐term effects in people's health. It provides drinking water while reducing the risk of disease for everyone, but specifically for children, pregnant women and older adults, who are some of the largest at‐risk populations in the Global South (Van Minh and Hung 2011). It also reduces other costs, such as the related expenditures in hospitalization, reduction in school absences for children and reduction in journeys to collect and carry clean water. However, point‐of‐use treatments are not commonly practiced due to a poor understanding of the link between the use of contaminated water and disease (Ogunyoku et al. 2011). Research shows that although relevant information helps people protect themselves against the risks of environmental contamination, such information disperses slowly by conventional means, such as formal education and media campaigns, and low‐income, uneducated households might be at a higher risk of misperceiving health risks of drinking contaminated water (Jalan and Somanathan 2008). There are other impacts of water quality increase as well, such as the change in odor and taste (Jeuland et al. 2016).

In this study, we use the theory of value of information (TVOI) as a means to influence investments in clean water (Wu and Zheng 2013). TVOI is broadly applied in various fields, for example, preservation of natural resources (Sheridan 2020; Wu and Zheng 2013), ecological protection (Keisler et al. 2014) and air pollution health preventive behaviors (Afridi et al. 2021). In our water purification study, the value of information depends on both uncertainty stemming from water quality (pollution) and the expected average health risks from drinking contaminated water. Value of information has been shown to be a significant influencer of behavior in several water resources management studies (Luoto et al. 2014; Bennear et al. 2013; Chen et al. 2007; Jalan and Somanathan 2008). Information, education and awareness programs decentralize the responsibility of mitigating behaviors to an individual level rather than the government (Zwane and Kremer 2007), and have been successful in reducing the risk of diarrhea (Gorham et al. 2017; Montgomery and Elimelech 2007). However, studies show that although water safety interventions are more effective than previously thought (Fewtrell et al. 2005), people in developing countries still have a poor understanding of the link between the use of contaminated water and disease (Joseph et al. 2016; Farham and Petro 2023), and although the value of information is large, households do not invest in information acquisition due to barriers such as lack of understanding of this link or knowledge of how to test for water quality. Therefore, providing information that is free and reliable (in our case, the results of the test on water quality and information on water purification options) could have a large impact on behavior.

Collective action‐based projects, such as community‐led total sanitation approaches have been shown to improve health outcomes of children in developing countries (Pickering et al. 2015; Turiansky 2021). Recent evidence, as demonstrated by Abramovsky et al. (2023), reveals heterogenous effects of sanitary interventions. For instance, these interventions exhibit greater efficacy in households where wives are involved in decision‐making (Augsburg et al. 2023; Meredith et al. 2013).

Our study is an informational intervention aiming at evaluating a change in behavior. There is substantial evidence that delivering salient information about household water quality increases the adoption of health preventive behaviors and investments in water quality (Brown et al. 2017; Madajewicz et al. 2007; Hamoudi et al. 2012; Lucas et al. 2011; Luoto et al. 2014; Trent et al. 2018). For example, Luoto et al. (2014) provide free safe water products to households while using framing and commitment marketing messages, and show an increase of water treatment product usage in Bangladesh and Kenya. Hamoudi et al. (2012) offer households water contamination tests and information on water purification methods, following up with them 1 month later. Using intention‐to‐treat methods where 88% of households in the treatment group were expected to have contaminated water, they find that households provided with a test and purification information were 5.3% points more likely to switch to using water from commercial suppliers.

In our study, we take a more laissez‐faire approach by assessing the contamination status of households' water and simply providing them with this information in one of our treatment conditions. In another treatment, we additionally provide information on water purification investments but refrain from supplying any sanitation products. We then compare the control group, which received no information about test results or purification methods, to these two treatment groups. This approach differs slightly from the study of Trent et al. (2018), conducted independently but during the same period as our study, which compares the effects of providing households with test results to offering community‐ and household‐level education sessions. By including a clean control arm, where we do not intervene, our study allows us to quantify the value of providing households with information. Compared to previous studies, our design focuses on estimating average treatment effects by comparing treatment and control groups specifically within the sample that had contaminated water, rather than using intention‐to‐treat methods. This allows us to directly measure the behavioral responses of households that are immediately affected by water contamination. Additionally, unlike more costly community‐led information and training sessions, our approach involves comparing the group that received basic information on water treatment methods to a group that received no such information. This simpler, lower‐cost intervention enables us to assess the effectiveness of providing minimal yet targeted information in driving behavior change, without the additional resource investment required for larger‐scale educational efforts.

We find that information on water contamination matters when aiming to change behaviors and lowering health risks of water‐borne diseases, and the short‐term impact is significant and large. Specifically, a few months after the intervention, 40% of the households provided with the water contamination test results and 48% of the households provided with the water contamination test results *and* information on water purification measures actually adopted water purification measures. This is compared to only 1.5% of the households adopting water purification measures when not given any information on water contamination or purification measures. The statistical likelihood of households adopting water purification measures controlling for a number of observable household characteristics was also close to our raw data‐based findings, at 38% and 51% in both treatments, respectively. Examining spillover effects within villages assigned to an information treatment alongside a control group of no such information, our study reveals that households not directly exposed to informational interventions exhibited minimal change in their purification behavior, even when their proximate neighbors demonstrated a higher likelihood for behavioral change. This underscores the significance of disseminating information pertaining specifically to the cleanliness of household water sources, rather than merely communicating general knowledge regarding the potential presence of E.coli in water or methods of purification. Examining heterogeneous treatment effects contingent upon household characteristics such as education, income, water access modalities, and recent encounters with water‐borne illnesses, our analysis reveals no statistically significant disparities. Additionally, considering the economic ramifications of diarrhea and the expense associated with water contamination assessment tests, our findings suggest that implementing straightforward purification methods, such as boiling water, could be a cost‐effective strategy to mitigate health risks. In the concluding section of our paper, we compare and discuss our results in the light of the results in the existing literature.

## 2 Study Settings

### 2.1 Experimental Design

**FIGURE 1:**
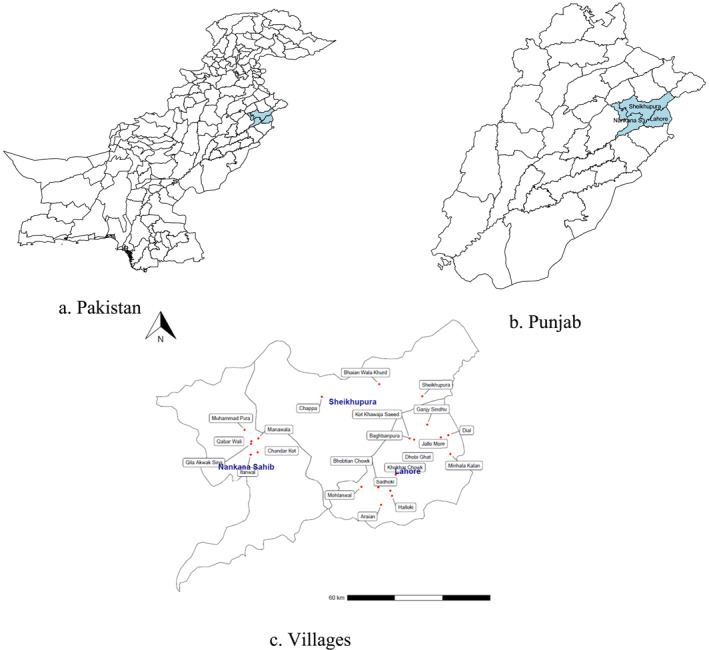
Location of districts and villages.

We conducted a semi cluster‐randomized control trial in 24 villages of the Lahore and Sheikhupura/Nankana Sahib districts in Punjab, Pakistan, to test water samples of households for the presence of bacteria of fecal origin. Figure 1 shows the map of the districts where we sample villages from. We employed a multistage random sampling technique to select the targeted households. In the first stage, we selected 24 villages (17 from Lahore and 7 from Sheikhupura/Nankana Sahib) purposefully. In the second stage, households were chosen to participate in the intervention. Households were selected from the list of village households using systematic random sampling techniques.

The study had three waves. Wave 1 of the study took place from October 2, 2017, to November 16, 2017, and from January 10, 2018, to January 17, 2018 (in Sheikhupura only), targeting a total of 1388 households. We gathered information on household demographics, socio‐economic conditions, source of drinking water, use of any water purification measure, episodes of diarrheal disease, and general awareness about health, sanitation, and environmental issues. At the end of the survey, we collected a sample of drinking water from household taps to test the water quality of households participating in the research, through E.coli water testing kits. Supporting Information S1: Online Appendix A includes the Wave 1 questionnaire.

Wave 2 was conducted 3 months after the conclusion of Wave 1, from February 15 to March 15, 2018. After the first wave where 1338 households were surveyed and their water tested, we found that only 379 of these had a drinkable water quality. These households were not part of the intervention, as their drinking water tested negative for E.Coli, meaning that their water was already “clean”. The rest of the households in this wave (around 73%) had fecal contamination in their drinking water. The households with water contamination were divided into three groups randomized on village‐level to receive one of the two treatments. Treatment group PT (partial treatment, 372 households in 9 villages) were presented with the results of the E.coli contamination test results (whether or not their drinking water has been tested positive for fecal contamination). Households in the partial treatment were contacted via phone regarding the water test results. Treatment group FT (full treatment, 309 households in 7 villages) were presented with the results of the E.coli contamination test and were given a one‐page handout containing information about in‐home water treatment options such as straining and boiling, use of disinfecting tablets/drops, electric and non‐electric filters, reverse osmosis, or change of water source (e.g., using bottled water instead of tap water). The full treatment households were contacted in‐person to deliver test results and the informational handouts. Supporting Information S1: Online Appendix B includes the handout given to households in Wave 2. During Wave 2, all 372 and 309 households selected for partial and full treatment, respectively, were reached, with no instances of refusal. Remarkably, many respondents proactively contacted the interviewers to inquire about their water test results. The number of observations for each treatment was similar to the studies using environmental interventions in randomized control settings such as Vu et al. (2020) and Carter et al. (2014).

The control group NT (no treatment, 328 households) was neither informed about their water test results nor given the hand‐out with information. The NT condition included 8 villages (198 households) where everyone was in a control condition plus an additional 130 households which were assigned to the control condition in the other 16 villages. These last 16 villages also had households assigned to either PT or FT. By having villages with only NT and also villages with both NT and PT/FT treatments, we are able to test for spillover effects between treatment and control within villages. Moreover, we can discern whether the provision of information solely regarding the presence of E.coli in water and purification techniques is sufficient to instigate behavioral change, or whether households must possess more tailored knowledge concerning the contamination status of their own water sources.

**FIGURE 2:**
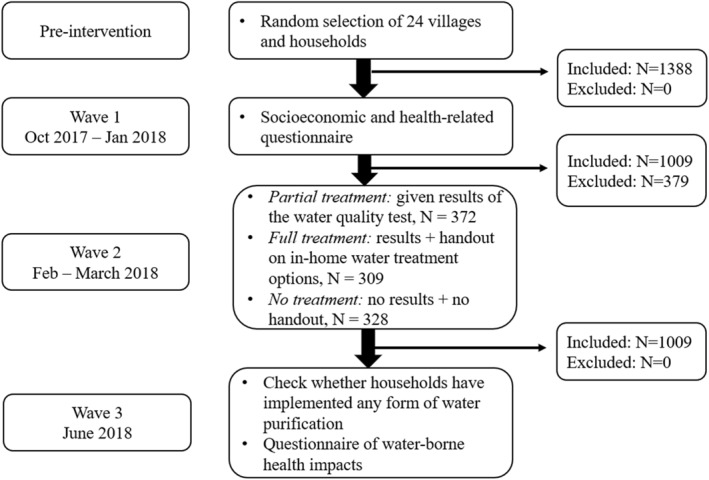
Flowchart of intervention.

In Wave 3 (June 2018), we visited the households and checked whether they had implemented any form of water purification at home. We surveyed all individuals with contaminated water in the PT and FT groups as well as the NT (control) group (total of *N* = 1009). Figure 2 shows a summary flowchart of the intervention. Supporting Information S1: Online Appendix C includes the Wave 3 questionnaire.

### 2.2 Randomization

The assignment of villages to treatments was randomized at the outset, as previously described in the experimental design subsection. Here we test whether the randomization was successful, in terms of observable characteristics of households being the same across the treatments. Table 1 shows the balance test of a number of socioeconomic, household and water and sanitation characteristics across the treatments measured at the baseline (Wave 1). For this we use the multiple hypotheses testing procedure of List et al. (2023) where the correlations between variables may create false positive test results.

**TABLE 1:** Balance table of observable characteristics across treatment arms.

	No treatment (control group)	Partial treatment	Full treatment	*p*‐values for test of equality		
				*NT* *=* *PT*	*NT* *=* *FT*	*PT* *=* *FT*
Socioeconomic characteristics						
Education of the household head (years)	4.06 (4.47)	5.08 (4.80)	5.07 (4.79)	0.03	0.05	0.98
Age of the household head (years)	47.15 (11.84)	47.33 (12.03)	48.52 (11.57)	0.95	0.49	0.76
People in the household	7.66 (3.04)	7.73 (3.20)	7.99 (3.77)	0.98	0.60	0.96
Monthly household expenditure (in pak rupees)	29,664 (14,948)	30,239 (14,557)	29,186 (16,683)	0.99	0.71	0.95
House characteristics						
Pucca (%)	72	82	81	0.01	0.05	0.94
Ownership (%)	97	94	87	0.36	0.00	0.14
Separate kitchen (%)	65	64	63	0.97	0.65	0.95
House surroundings rated dirty by the assistant (%)	34	34	38	0.98	0.68	0.94
Water and sanitation						
Already purified drinking water (%)	5	6	8	0.97	0.21	0.80
Plastic water storage (%)	76	90	93	0.00	0.00	0.93
Water accessed from tap (%)	54	47	44	0.52	0.21	0.97
Toilet discharges to a river or drain (%)	23	22	25	0.99	0.79	0.95
Family member suffered from diarrhea in the last month (%)	28	29	31	0.98	0.35	0.91
Health knowledge						
Diarrhea patients should use more water (%)	48	55	53	0.42	0.76	0.98
Patients' water should be treated/filtered (%)	70	63	58	0.41	0.01	0.87
*N*	328	372	309			

*Note:* Standard deviations in parentheses. NT stands for No treatment (control group) PT/FT stands for Partial/Full treatment. *p*‐values are corrected for multiple hypotheses testing procedure introduced in List et al. (2023). The variables were measured in the Wave 1 only.

As shown in Table 1, there are several characteristics that differ between treatments. This is unfortunately by chance. For example, although the difference is not extreme, education of the household head in the treatment groups is around 1 year longer on average than the control. At the same time, treatment groups have a larger percentage of pucca houses (i.e., dwellings that are designed to be solid and permanent) than semi‐pucca or kutcha houses (dwellings made of mud and straw) compared to the NT group. There are also some differences in the percentage of houses owned versus rented and water storage facilities and household's prior knowledge of the need for water treatment in the case of patients with diarrhea. Given these differences between treatments, our empirical analysis to explore the impact of the treatments on the decision to change water purification will mainly focus on the regression analysis with household‐level controls. We will also assess the heterogeneity in treatment effects according to households' education level, income, water access and if a family member has been recently affected by diarrhea. Our main variable of interest, whether households have been purifying their drinking water pre‐intervention shows no significant difference between the treatments: 5%, 6% and 8% of households purified their drinking water pre‐intervention in the NT, PT and FT treatments, respectively. Almost all of these households (98%) was purifying their water using the boiling method.

Supporting Information S1: Table E1 in the Online Appendix shows the balance table with a similar testing of across treatment differences in observable characteristics when we only focus on the villages that had households that were assigned either to the control (NT) and treatments (PT/FT) within the same village. We do not observe any difference in observable characteristics within this sample between the partial/full and No treatment conditions. Hence, we will use the analysis based on this sub‐sample as a robustness test of our main results. Results for this subsample will be reported in the Online Appendix, unless specifically stated.

### 2.3 Measuring Water Contamination

We tested household water for fecal contamination using E.coli kits developed locally (the test was branded HydroCheck during the intervention but later changed its name to SinoW). The E.coli test kit was created by the Department of Chemistry, COMSATS University (see the Supporting Information S1: Online Appendix D for a photo of the test). This product is approved by the government and is commercialized by COMSATS, being available in many medical stores and pharmacies. A complete user manual is attached with the packet of the product (see Supporting Information S1: Online Appendix D). The product packet includes a small, sealed bottle containing the chemical to test the water, a syringe to inject the water sample into the sealed bottle, and a user manual. In the first step, the project team fills the syringe from the drinking water source. This reduces potential contamination of the water during handling. Then, the water sample is injected into the sealed bottle for testing. The product bottle is shaken well and stored in a cool place for 24 h. After 24 h, the team checked the color of the water inside the sealed bottle. If the color of the water turns yellow (the original color of the water is purple), it means that the water is contaminated and thus, cannot be drank. If the color of the water in the sealed bottle remains the same (purple), the water is not contaminated and thus drinkable.

## 3 Empirical Strategy

To examine the impact of the water testing and information provision treatments, we take a three‐step approach. First, we test for differences in the percentage of households adopting or changing water purification. Specifically, we perform non‐parametric tests of the hypothesis whether households implemented their form of water purification between the treatments (partial/full treatment vs. no treatment) and whether there were significant differences in water purification implementation when an informational component is added to the water test results (partial treatment vs. full treatment). Second, we predict the likelihood of water purification change by the household post‐intervention. For this, we estimate models that include a set of dummies for the treatments, as well as household level controls such as socioeconomic characteristics, household characteristics and water and sanitation characteristics. We provide outputs for both ordinary least squares (OLS) and probit estimations (probit reported in the Supporting Information S1: Online Appendix E). The general model is as follows:

$$∆WaterPurification_{i}=\alpha_{0}+\alpha_{1}PT_{i}+\alpha_{2}FT_{i}+\alpha_{j}\sum_{j}Z_{j}+\epsilon_{i},$$

Where ∆ *WaterPurification* equals one if the household has implemented water purification measures, PT and FT variables are dummies where 1 = partial treatment and 1 = full treatment respectively (with the omitted category of no treatment, NT), $Z_{i}$ are the socio‐economic characteristics, house characteristics, water and sanitation controls reported in Table 1. In our Wave 3 questionnaire, we specifically asked whether households had made any changes in the water purification methods used, and if so, what were the changes made. We present the results with errors clustered at village level.

We test the robustness of the results using alternative model specifications using probit regressions and propensity score matching which are presented in the Online Appendix. Propensity score matching allows us to estimate the causal effect of the treatments (PT/FT vs. NT) by accounting for heterogeneity and gaining precision on our estimates (Angrist and Pischke 2009). We also present the analysis of how heterogeneity in characteristics affects the implementation of the three treatment arms (in a separate section). For this, we provide marginal effects for selected socioeconomic characteristics, household types and water and sanitation features.

Although the intervention was not directly designed to test this, it does allow us to provide at least a lower bound for cost‐effectiveness calculations in terms of averted costs of the implementation of the intervention. For this, we follow the process used by Kremer et al. (2023) for a water purification cost‐effectiveness analysis in Kenya (using chlorine dispensers), which is an example of a cost‐effective reduction in child mortality and use various assumptions on the cost of water purification.

As the intervention was not formally pre‐registered in 2017, the results in this manuscript are exploratory and should be supplemented with further work. A non‐formal pre‐registration of the study was approved by the grant provider Higher Education Commission of Pakistan in May 2017, outlining the design of the intervention and hypotheses to be tested and the analysis plan. These documents (along with the ethical approval for the intervention) were submitted as part of the journal's review process but are not included in the Online Appendix due to the grant providers' confidentiality policies.

## 4 Results

### 4.1 Non‐Parametric Testing of Treatment Effects

**FIGURE 3:**
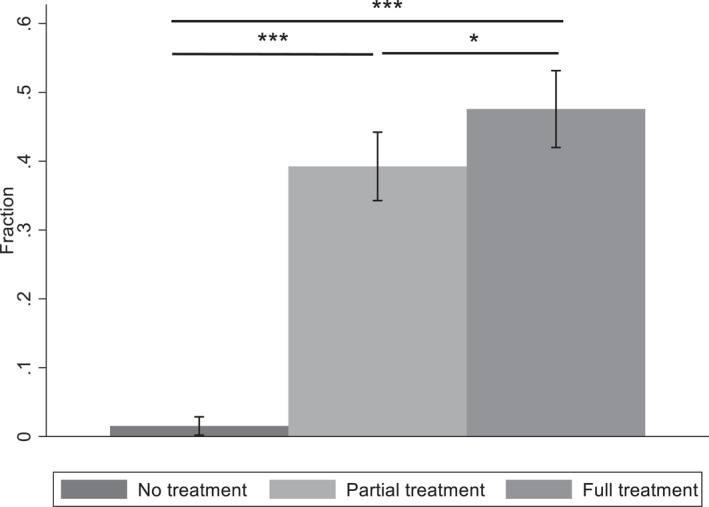
Percentage of households in each treatment arm that implemented water purification measures after Wave 3 (June 2018). Error bars are 95% confidence intervals. *p*‐values of percentage tests (horizontal lines represent the comparisons between: NT vs. PT, NT vs. FT and PT vs. FT): *p < 0.10, **p < 0.05, ***p < 0.01.

Figure 3 summarizes the percentage of households who have adopted new water purification methods in each intervention arm, compared with the information obtained in Wave 1 (our baseline). We find that 6.5% (59 out of 901 households who answered this question voluntarily in the baseline—from the 1009 that comprise the total sample) of the households which were using water purification methods in the baseline. Given the number of households already purifying water in the baseline, we consistently use the term “implemented water purification measures” when generalizing the adoption of water purification.

For the case of the NT condition (control), we do not expect households to have changed their behavior, as these households were not provided with any additional information. In this group, only 1.5% of households implemented water purification measures, which accounts for a small group of households deciding to start purifying their water without any water test results or information provision. Consistent with our expectations, in the PT around 40% of households implemented water purification measures, while in the FT the percentage went up to around 48%. A non‐parametric $\chi^{2}$ test, for treatment differences shows that the proportion differences between NT versus PT, and NT versus FT are significant (*p‐value = 0.000*), as well as the difference between PT versus FT (*p‐value = 0.029*).

In terms of the type of water purification method, in the PT (FT) around 41% (46%) of households started boiling their water, 19% (27%) used a government‐provided, non‐electric filter, 15% (20%) asked their neighbors for clean water, 8% (6%) started using bottled water and another 2% (2%) used electric filter. In the PT treatment another 13% used alternative methods such as started purchasing non‐bottled clean water, changing hand‐pumps or collecting water from other clean sources. When we test for the distribution of purification methods being equal across the treatments we find that households in the PT treatment use more diverse purification methods than the ones in the FT treatment (*p‐value = 0.000*). If we remove the 13% of households in the PT treatment which changed hand pump or started purchasing or collecting water from other sources, we find no significant difference between PT and FT treatments in the proportion of those that started to boil water, used electric or government filters, bought bottled water or asked neighbors for clean water (*p‐value = 0.525*).

As shown in Figure 3, there were no average spillover effects going from the treated households to those in the control, regardless of whether we had villages with more than one intervention arm. Less than 2% of households implemented water purification measures in the control. To formally test for spillovers, we compare the households in the NT in the villages with and without multiple intervention arms. We find that the change in behavior was the same (1.6% vs. 1.4%, $\chi^{2}$ *p*‐value = 0.859). Hence, we can rule out any spillover effects and can confidently pool the data for all NT households when presenting the results.

### 4.2 Regression Analysis of Household Purification Behavior

Table 2 presents the results from OLS regressions predicting the household decision to implement water purification measures following the intervention, for the full sample and the sub‐sample with both treatments and control. Models 1 and 3 report the results without any inclusion of controls while models 2 and 4 include the controls that are reported in Table 1. By controlling for these variables, we make sure that any possible assignment differences across the treatments are accounted for, particularly those which lead to an imbalanced sample (see Table 1). Similar to the results in Figure 3, the results in column 1 show that in the NT only around 1.5% of individuals are estimated to implement water purification measures. Meanwhile in the PT and FT the estimated percentages are 40% (0.377 + 0.015) and 48% (0.460 + 0.015) respectively. Note that, the estimated treatment effects change when we adjust covariates in the alternative regression models. In Models 3 and 4, we test whether the results are robust to restricting sample to those villages where there were multiple intervention arms within a village. The results are consistent with the results from the full sample.^1^

**TABLE 2:** Predicted implementation of water purification measures, post‐intervention.

	All villages		Villages with treatments and control	
OLS regressions	(1)	(2)	(3)	(4)
Partial treatment	0.377***	0.393***	0.382***	0.383***
(Water test)	(0.032)	(0.031)	(0.039)	(0.033)
Full treatment	0.460***	0.501***	0.428***	0.455***
(Water test + information)	(0.040)	(0.046)	(0.037)	(0.043)
Socioeconomic controls				
Education HH		0.006		0.005
		(0.004)		(0.005)
Age HH		0.000		−0.000
		(0.001)		(0.001)
HH Size		−0.008**		−0.009*
		(0.004)		(0.005)
HH Expenditure		0.001***		0.000***
		(0.000)		(0.000)
House controls				
Pucca		−0.012		−0.035
		(0.034)		(0.050)
Rented		−0.100**		−0.089*
		(0.037)		(0.045)
Separate kitchen		0.020		0.041
		(0.031)		(0.042)
House surrounding dirty		0.022		0.018
		(0.029)		(0.042)
Water and sanitation controls				
Plastic water storage		0.021		−0.026
		(0.109)		(0.131)
Metal storage		0.164		0.213
		(0.129)		(0.166)
Water tap		0.022		0.030
		(0.022)		(0.029)
Toilet discharge		−0.019		−0.020
River/Drain		(0.035)		(0.046)
Recent diarrhea		−0.030		−0.005
		(0.033)		(0.029)
Health knowledge controls				
Diarrhea patient drink more		−0.021		−0.047**
		(0.017)		(0.020)
Patients' water treated		−0.019		0.028
		(0.031)		(0.031)
Constant	0.015***	−0.183	0.014	−0.119
	(0.005)	(0.176)	(0.009)	(0.225)
*Chow test p‐value PT* *=* *FT*	*0.108*	*0.037*	*0.354*	0.128
Observations	1009	1002	712	705
*R* ^2^	0.187	0.241	0.119	0.190

*Note:* No Treatment (Control condition) is the omitted category for treatment comparisons. Partial treatment (PT: water contamination results = 1) and Full treatment (FT: water contamination results + information provided = 1) are included as explanatory variables. Standard errors clustered at village level. Controls include all the variables described in Table 1. HH stands for Household.

^*^ *p* < 0.10.

^**^ *p* < 0.05.

^***^ *p* < 0.01.

Next, we conduct a multinomial logistic regression analysis to test whether the purification methods adopted differed between conditions (Table 3). Households were asked to specify the type of purification method they adopted after indicating if they had implemented a new method. However, not all households provided details on the specific method used, so our analysis is limited to those who reported this outcome. For example, none of the 1.5% (5 households) in the NT condition specified their method of purification. Therefore, our analysis focuses on comparing the PT and FT conditions, where households did implement and specified a method of purification (almost 700 households). We do not find any systematic differences in the propensity to not report the purification method based on household characteristics, except for the number of household members and expenditure levels. As expected, households with more members have more missing values, while higher education levels are associated with fewer missing values. We thus include all control variables as reported in Table 1 in the multinomial logistic regression analysis. Table 3 presents the results of this analysis reporting the marginal effects of predicting the likelihood of adopting the purification method as compared to the alternative treatment. We find that, compared to the base category of using “other” purification methods, households in the FT condition are significantly more likely (+25% points—pp) to use boiling than those in the PT. Similarly, they are significantly more likely (+17pp) to use some form of electric filter (whether government‐provided or privately purchased) compared to the PT households. In contrast, households in the PT condition are more likely (+76pp) to adopt other types of purification methods, as reflected by the constant coefficients in the regression model. This finding aligns with the non‐parametric test results discussed in the previous subsection.

**TABLE 3:** Multinomial logistic prediction of the purification type (in the PT and FT conditions).

	(1)	(2)
Purifying through filter		
Full treatment	0.172***	0.178***
	(0.215)	(0.015)
Purifying through boiling		
Full treatment	0.254***	0.260***
	(0.025)	(0.025)
Purifying trough other type		
Constant partial treatment	0.755***	0.763***
	(0.022)	(0.021)
Constant full treatment	0.576***	0.561***
	(0.014)	(0.028)
**Controls**	**No**	**Yes**
Observations	681	675
Pseudo *R* ^2^	0.022	0.082

*Note:* Marginal effects of predicted likelihood of adopting purification methods are reported. Robust delta method standard errors are in parentheses. Other purification types is the base category. No Treatment condition is omitted because of almost no purification change was observed in this condition. Controls are the variables used in Table 2. Filter category contains both using privately bought electric filters and government provided ones.

*p < 0.10.

**p < 0.05.

^***^ *p* < 0.01.

Lastly, as we indicated in the informal pre‐registration document, to control for heterogeneity between households and treatment, we conduct a propensity score matching to estimate average treatment effects on the change in purification method, using socioeconomic, household, and health/sanitation covariates as nearest neighbors. We match households separately for each covariate group, as including all covariates does not yield any matches. The results are reported in Supporting Information S1: Table E3 of Supporting Information S1: Online Appendix E. The treatments produce significant average effects with a smaller number of nearest neighbor matches, consistent with our findings from non‐parametric and regression analyses.

### 4.3 Heterogeneity Analysis of Household Purification Behavior

In this section we examine how different types of households react to the treatment conditions (partial and full treatment) compared to the no treatment and whether each treatment condition is more effective on certain household characteristics. Supporting Information S1: Table E4 in the Supporting Information S1: Online Appendix E summarizes the tests for heterogeneous treatment effects with the inclusion of controls. More specifically, we investigate whether the effects are different by education of the household head (being above or below a median of 5 years of education), household expenditure (being above or below median expenditure of 25,000 Pak Rupees), whether water is access through a tap (compared to no tap) and whether the household has recently experienced a case of a diarrhea among any of the members (or not).

**FIGURE 4:**
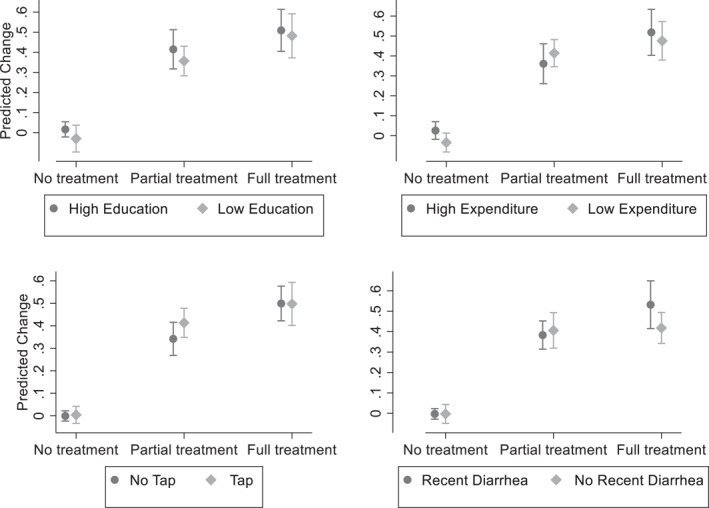
Marginal effects from heterogeneity analysis of treatment effects to predict the implementation of water purification measures (Full Sample). Effects come from the regressions reported in columns (1–4) of Supporting Information S1: Table E4 in the Online Appendix. Error bars are 95% confidence intervals.

We present the marginal effects of the heterogeneity‐based regressions in Figure 4 by household type and treatment condition, which complements the results from the Supporting Information S1: Table E3 in Online Appendix. We find no differential effects of the treatments by household characteristics. Supporting Information S1: Table E5 in the Online Appendix reports the results for the reduced sample of villages where households were assigned to both partial/full and no treatment conditions. The results mirror the full sample results. The only difference are those households with high expenditure have smaller differences between the treatments and control than those with low expenditure. This is driven by high expenditure households changing their water purification significantly more than low expenditure households in the control NT condition. However, given that only two households changed their purification methods in the NT condition, we interpret this finding with caution as these were the outliers. These two households happened to be wealthier than the remaining 142 households who did not change their purification methods in the NT condition in the villages where both treatment and control were present.^2^

As an extension to the heterogeneity analysis, by examining responses from the Wave 1 (pre‐intervention) questionnaire we focus on how the provision of information could help align prior knowledge on clean water and diarrhea and use this to leverage the change in water purification behavior. Table 4 shows the percentage of households in each treatment arm who respond to the questions “What do you think causes diarrhea?” and “What do you think you should do to prevent diarrhea in your household?“. Most households demonstrate good knowledge of the causes of diarrhea, including water and food. Over 60% of households mention water in their responses and one third mention food as a cause. Moreover, over a third of the sample know that water changes can prevent diarrhea, yet they do not practice water purification measures. Many households also mention “clean” water and food as preventive measures without implementing purification measures. Therefore, there could be a link between providing information on water quality and promoting water purification in our treatments to emphasize and leverage prior household knowledge.

**TABLE 4:** Responses to questions on diarrhea knowledge (%).

What do you think causes diarrhea?	No treatment	Partial treatment	Full treatment
Water	64%	63%	65%
Food	27%	32%	36%
Not washing hands	0%	1%	1%
Other	12%	12%	6%

**What do you think you should do to prevent diarrhea in your household?**	**No treatment**	**Partial treatment**	**Full treatment**
Water	37%	36%	30%
Cook food	1%	1%	0%
Hand washing	1%	1%	2%
“Clean” water and food	16%	16%	27%
Households in treatment arm	328	372	309

Table 5 extends the specifications presented in Table 2, adding these knowledge variables (using an OLS approach). We find that knowing that water is a direct cause of diarrhea increases the likelihood of implementing water purification measures, regardless of the information provided (either only results from the water quality test—PT—or results from the test plus information sheet—FT). Controlling for the knowledge variable in the regressions, the likelihood of implementing water purification measures is 39% for households in the PT and 50% for households in the FT, while it slightly drops to 34% and 40% respectively for the model with interactions. It does not significantly affect the likelihood of implementing water purification measures for those who are not provided information on water quality, but it does have a significant effect on those who are provided information. In the model with interactions, we also find that households with the baseline knowledge of water as a cause of diarrhea respond to the PT treatment more than those without such knowledge, but the effect is marginally significant: PT is 16pp more likely to implement water purification measures.

**TABLE 5:** Knowledge about the link between water quality and diarrhea predicting implementation of water purification.

	(1)	(2)
Partial treatment	0.394***	0.341***
	(0.031)	(0.054)
Full treatment	0.501***	0.395***
	(0.046)	(0.090)
Cause of diarrhea: Water	0.076**	−0.009
	(0.034)	(0.026)
Water × partial treatment		0.164*
		(0.095)
Water × full treatment		0.084
		(0.060)
Cause of diarrhea: Food	−0.001	0.001
	(0.025)	(0.026)
Prevention channel: Water only	0.010	0.017
	(0.038)	(0.038)
Prevention channel: Clean water and food	0.003	0.009
	(0.040)	(0.039)
Constant	−0.230	−0.175
	(0.171)	(0.178)
Observations	1002	1002
*R* ^2^	0.248	0.253
Controls	Yes	Yes

*Note:* Standard errors clustered at village level from OLS regressions are in parentheses. No treatment is the benchmark group. Heterogenous treatment effects. Same set of controls are used in all regressions as in Table 1.

^*^ *p* < 0.10.

^**^ *p* < 0.05.

^***^ *p* < 0.01.

### 4.4 Cost‐Effectiveness Analysis

Given the results of the intervention, we aim to provide an illustrative example of a cost‐effectiveness analysis based on the water treatment information intervention used in this study. The calculations follow Kremer et al. ’s (2023) example for chlorine dispensers in Kenya (by Evidence Action 2019), which is an example of a cost‐effective reduction in child mortality in low and middle‐income countries. For our calculations, we use several assumptions. First, we assume that our intervention reduces but not eliminates diarrhea. Second, and following Kremer et al. (2023), we assume that the intervention targets diarrhea reduction in children under 5 years of age, leaving the benefits from other groups aside.

We examine cost‐effectiveness using the following assumptions. First, we assume that the intervention increases the number of households boiling water, which is the change that most of the treated sample in our study chose as their form of water purification (40% and 45% of the sample for the partial and full treatments). Boiling is the most commonly used and reported point‐of‐use household water treatment globally (Rosa and Clasen 2010), compared to other options like chlorination. Although not a perfect method, several studies show that boiling water could help reduce under 5 mortality (and local populations have knowledge of this relationship between treated water and diarrhea in children), especially in regions with poor water quality, as it prevents waterborne diseases, while killing pathogens at a low cost (Fagerli et al. 2017; Floess et al. 2024; McLennan 2000; Li and Xiao 2023).

In the first scenario, we assume that the cost of boiling water per year, in 2023 values is USD 44.5 per household per year (USD 223 per 5 years), where each household in our sample that has at least 3 people (we have 679 of these in our sample, compared to 681 households treated total) has a child under 5. This value comes from the calculations of T. Clasen et al. (2008) for India, updated by inflation for 2023. In a second scenario, we use the average monthly cost of boiling water reported by households for “average” and “good” water quality (USD 0.29 or PKR 188/month, USD 19/5 years). Lastly, in a third scenario and to have a range of potential water boiling cost values, we use the maximum monthly cost of boiling water reported by households in the intervention paying for “average” and “good” water quality (USD 8.64 or PKR 5253/month, USD 518/5 years). For the purposes of analyzing the scalability of the intervention, we include the cost of the water test kit per year, which is now sold in pharmacies and markets around Pakistan at PKR 300 per test (USD 3.63/USD 18.2 5 years). In addition, and following T. Clasen et al. 2008, we include the average indirect costs of boiling water, assuming each household uses 6 L of boiled water per day (USD 180/5 years). This cost is already included in the cost of boiling water in T. Clasen et al. 2008. Boiling water, as with any other water treatment, is a burden to households, but mainly lands on women and girls (Cherukumilli et al. 2023; T. Clasen et al. 2008; Ray 2007). To make all scenarios comparable, we use monetary values expressed in 2023 USD values.

To calculate the cost per disability‐adjusted life year (DALY) averted, we use the recommendation of the World Health Organization (World Health Organization 2001; Murray et al. 1994) assumption that a year of life in the first 5 years of life is equivalent to 81.25 DALYs and an average death age of 2 years old. In the case of the expected DALYs averted per person, we follow the meta‐analysis calculations of Kremer et al. (2023) and the approach outlined by the World Health Organization (2020). To estimate the cost per DALY averted we take the quotient of the estimated cost per under 5 by the expected number of deaths averted, then divide the cost by 81.25. A step‐by‐step explanation of how the final measure of cost per DALY averted was calculated can be found in the note in Table 6, including the assumptions used.

**TABLE 6:** Cost‐effectiveness analysis (in 2023 USD, cost of boiling water).

		Cost of boiling water from T. Clasen et al. (2008)	Average water purification cost reported by households	Maximum water purification cost reported by households
Costs				
(1)	Cost of boiling water/5 years	223	19	518
(2)	Ccost of water test kit/5 years	18.2	18.2	18.2
(3)	Cost of time (opportunity cost of time used for boiling water)/5 years^a^		180.0	180.0
(4)	Total cost/5 years	241.2	217.2	716.2
Assumptions from Kremer et al. 2023, evidence action				
(5)	Mortality rate	0.069		
(6)	Posterior predictive mean risk ratio of effect	0.77		
(7)	Average effective compliance in meta‐analysis	0.53		
Assumptions from WHO				
(8)	DALYs from death in the first 5 years	81.25		
Data and calculations for water purification intervention				
(9)	Effective take up rate for intervention	0.44		
(10)	Effective deaths averted per person	0.0132		
(11)	Expected DALYs averted	1.070		
(12)	Cost per death averted	18,303.5	16,481.9	54,356.3
(13)	Cost per DALY averted	225.3	202.9	669.0
Comparison thresholds				
(14)	GDP Pakistan 2023	1407		
(15)	3 × GDP	4221		
(16)	0.5 × GDP	703.5		
(17)	WHO‐CHOICE average cost (37 interventions + 12 packages, USD per DALY)	337.4		
(18)	WHO‐CHOICE cost for intervention MNCH_33.ORSzinc (USD per DALY)	22.3		

^a^ Indirect costs of T. Clasen et al. (2008) are already incorporated in the total cost of boiling water. Total cost (4) is the sum of the cost of boiling water (1), the water test kit (2) and the opportunity cost of time (3). We take the conservative assumption for the mortality rate (5), using the expected mortality rate from the chlorine dispensers intervention from Kremer et al. (2023) and Evidence Action (201), as well as the (7) effective compliance in the meta‐analysis of 53% and the (6) posterior predictive mean risk ratio of the effect, which the authors convert from a predictive distribution of odds ratios, assuming the prior mortality rate. We assume that a (8) death within the first 5 years of life leads to 81.25 DALYs (Murray et al. 1994, World Health Organization). Stenberg et al. (2021) uses DALY data to calculate the cost‐effectiveness ratio, but shows the value as a ratio of Dollars/Healthy Life Years. (9) comes directly from our water purification study as the average take up of partial and full treatments ‐ the take up in the control (no treatment). The (10) effective deaths averted per person = (5)*[(9)/(7)]*[1‐(6)]. The (11) expected DALYs averted = (10)*(8). The (12) cost per death averted = (4)/(10) and the (13) cost per DALY averted = (12)/(8) for each of the three scenarios considered.

In addition, to compare the cost‐effectiveness of the intervention, we use the Pakistani GDP per capita, which is USD 1407 in 2023 (World Bank Databank 2024). The GDP per capita is the threshold suggested by the Commission on Macroeconomics and Health of the World Health Organization (World Health Organization 2001; Bertram et al. 2016; Opryszko et al. 2010) to conclude whether an intervention is highly cost‐effective or not. We also compare other thresholds, such as 3 times the GDP for a “cost‐effective” intervention, and 0.5 times the GDP for a “opportunity cost” threshold (Kazibwe et al. 2022; Marseille et al. 2014), as well as against the latest WHO‐CHOICE estimates for maternal, newborn and child health in Eastern Sub‐Saharan Africa and South‐East Asia for intervention #33, *Management of diarrhea through oral rehydration solution and zinc* (MNCH_33.ORSzinc) and the average values of the 37 interventions + 12 programs used (Stenberg et al. 2021).

Table 6 presents our cost‐effectiveness calculations for the case described above. Within the intervention, the cost per expected DALY averted is consistently lower than the Pakistani GDP per capita for any of our three scenarios. For example, for the maximum water cost scenario, the cost per expected DALY averted is USD 669, compared to the GDP per capita of USD 1407 and still lower than half of the GDP per capita threshold. In the case of the cost of boiling water from T. Clasen et al. (2008), the cost per expected DALY averted is USD 225.3, which is still lower than any of the GDP per capita thresholds used. The average water purification cost per DALY averted is lower than the GDP thresholds and the average WHO cost effectiveness measure (USD 202.9 vs. USD 337.4). However, our maximum cost per DALY averted is higher than the WHO‐CHOICE estimates (USD 669 vs. USD 22.3 for MNCH_33.ORSzinc and USD 337.4 average; Stenberg et al. 2021). Given this, we conclude that our intervention was cost effective for three out of the four measures included. Specifically, the intervention was consistently cost effective against the local GDP measures, but that is not the case against the WHO measures.

## 5 Concluding Discussion

Risk communication can be used as a low‐cost strategy in the battle against water‐borne diseases, given that decisions on water purification investments are made sporadically while providing long‐term health benefits. One of the most pressing issues in terms of water quality is how best to convey risks in ways that individuals can clearly understand and use to make long‐term changes to their water quality. In this study, we attempt to show how targeted information on water quality at the household level positively affects the demand for water purification in Pakistan. We use two low‐cost interventions, the provision of information about the results of a water contamination test and the provision of information on potential investments in water purification to increase household changes in water purification measures.

In almost three quarters of our initial sample of 1388 households, we saw evidence of fecal contamination (E.Coli) in their drinking water. However, the result of our randomized controlled trial suggests that campaigns that include helpful information can make a significant and positive impact on people's decisions to implement water purification measures. While only 1.5% of households assigned to a no‐information treatment implemented water purification measures, 40% of households provided with water quality test results (PT) and 48% of households provided with both water quality test results and information (FT) did so. More information in this case, means more households changed their behavior in relation to water quality at almost no additional cost to the information provider (assuming that the information compiled on water purification measures is sunk and has zero marginal cost).

This is evidence that individuals and households will engage in health‐promoting activities when they are aware of the associated risk. Even one‐time targeted information of the type we used in our trial can have long‐term implications for public health and its associated costs. More particularly, household members can be educated about hygiene and the health risk of poor‐quality drinking water by an appropriate information campaign. This is a cost‐effective way of achieving the UN SDG 6 and reducing the disease burden in Pakistan through an intervention which could be applied in similar developing countries.

Nevertheless, our short‐timed intervention to at‐home water purification is not perfect: around half of the households in the experiment continued to drink contaminated water a few months after the intervention. However, it is encouraging to note that while in the very short timeframe (1 month wait between intervention and data collection on water purification measures), many households did not make changes to their water quality, some households were planning to do so in the future. More specifically, we asked a question on planned change in purification to the respondents from the partial and full information treatments who had not implemented any purification measure. More than 80% of this subsample stated that they were planning to implement water purification measure in the future.

Our results can be directly compared to those of Trent et al. (2018), who conducted their study during the same period. They found that providing households with water contamination test results led to significant improvements in water quality and water treatment behaviors. Similar to our findings, they observed that households reported using simple water treatment methods, such as boiling, 42% points more often in the treatment group where test results were provided. On the other hand, providing households with community‐ and household‐level training sessions had a very limited effect on water quality and treatment behaviors. While water quality did not improve, there was a small 3% points increase in households' reported water treatment. Our results align closely with these findings: test result information produced the most significant change in water treatment behavior, while additional information on treatment methods only slightly increased purification behavior. This suggests that general information campaigns, such as community‐level training or social media campaigns, may yield limited results in driving behavior change. Instead, policymakers should consider targeting households with more direct and specific information about their water, for example, by providing test kits to encourage meaningful behavior change.

One caveat of our research design was that our main outcome variable, the change in at‐home water purification, is self‐reported, which could have some degree of experimenter demand effect toward the enumerator (Zizzo 2010). While we cannot directly assess the impact of experimenter demand effect on responses, we can mitigate this concern by conducting further analysis. This involves examining responses to follow‐up questions regarding whether households have made changes to water purification. Enumerators were instructed to document the details of the indicated changes in two open‐ended questions (see Online Appendix C). Responders in the Full treatment condition had access to suggested purification methods from Wave 2 handouts, making their responses more likely to be biased by experimenter demand effects. However, those in the PT condition lacked such information. Consequently, we would anticipate households in the PT condition to struggle in responding to open‐ended questions unless they have implemented the indicated purification method.

Our results indicate that although a one‐time targeted information package of this cluster‐randomized control trial can have sizable effects on mitigating behavior, repetition of information campaigns could help increase the number of households implementing risk‐reduction measures. Repetition could reinforce the understanding that water test results are not random. Households that have not started purification may recognize that their water remains contaminated, while those already purifying their water may gain reassurance that their efforts are effective, encouraging continued purification. Therefore, more regular water testing campaigns in short intervals and appropriate information packages can help mitigate unsafe drinking water, at least in our target area. In addition, more research is needed on how additive interventions can be used to reduce the impact of water‐borne diseases, including specific types of water treatment measures, and given that boiling water is the most common change made by households in this study. For example, using other low‐cost, point‐of‐use safe water products such as chlorine or a filter where technologies remain unused, and specifically for the global poor (Haushofer et al. 2021; Luoto et al. 2014, 2011).

## Ethics Statement

The ethical approval was granted by DARC at COMSATS University.

## Conflicts of Interest

The authors declare no conflicts of interest.

## Declaration of Generative AI and AI‐Assisted Technologies in the Writing Process

Generative AI and AI‐assisted technologies have only been used during the writing process for the purposes of proofreading the manuscript.

## Supporting information

Supporting Information S1

## Data Availability

The data that support the findings of this study are available on request from the corresponding author. All data will be publicly available with the replication package upon acceptance of the paper, including survey instruments.

## Endnotes

^1^
In addition, as a robustness test, Supporting Information S1: Table E2 in the Online Appendix reports the results using probit regressions as indicated in the pre‐registration document: the results are statistically and qualitatively similar to that of OLS regressions. Supporting Information S1: Figure E1 in the Online Appendix presents the marginal effects for the full sample specifications (from Table 2 and Supporting Information S1: Table E2). These marginal effects represent the probability (likelihood) that the household implemented water purification measures and is in line with the discussion presented above.

^2^
Alternatively, we also include the raw proportion of households starting water purification in each treatment by the subgroups in the Supporting Information S1: Figure E2 of the Online Appendix. The results are consistent with the ones from the regression analysis with an exception that in the PT, we find that higher expenditure households change their water purification more than the lower expenditure households. This result is also consistent with the regression results reported in the Supporting Information S1: Table E4 in the Appendix: PT condition is more effective on high expenditure households than low expenditure households.
