# Supplementary material for: Clear Waters, Bright Futures: Do Low‐Cost Information Interventions Increase Health Preventive Behaviors
Source: Health Econ. 2025 May 20;34(9):1578–94. doi: 10.1002/hec.4977 (PMC12316582; doi:10.1002/hec.4977)
Supplement: Supplementary file 1 — Supporting Information S1 [file HEC-34-1578-s001.docx]

**Online Appendix for**

**“Clear Waters, Bright Futures:**

**Do Low-Cost Information Interventions Increase Health Preventive Behaviors”**

Appendix A: Wave 1 questionnaire

Appendix B: Handout given to households in Full treatment, Wave 2

Appendix C: Implementation of water purification measures questionnaire, Wave 3

Appendix D: Photos and instructions of the E.coli test kits (HydroCheck / SinoW)

Appendix E: Supplementary figures and tables

# Appendix A: Wave 1 questionnaire


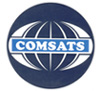


**COMSATS Institute of Information Technology, Lahore**

*Department of Management Sciences*

_________________________________________________________________________________________________________

**Household Survey on Water Quality**

**Researchers: Dr. [[[hidden for blind review]]] and Dr. [[[hidden for blind review]]]**

My name is_________ and I am from COMSATS Institute of Information Technology, Lahore. This survey is conducted for the above research study by Dr. [[[hidden for blind review]]] and Dr. [[[hidden for blind review]]] from COMSATS Institute of Information Technology, Lahore. The purpose of this survey is to collect household water quality data for research purposes. Please answer the questions to the best of your knowledge. Answers will be kept completely confidential and will only be presented in a summary format. Your cooperation in this survey by way of providing accurate information is highly appreciated and duly acknowledged.

**_______________________________________________________________________________________________________________________**

Survey Date [_ _ /_ _ /_ _] Start time [_ _ /_ _] End time [_ _ /_ _]

Name of the village/area (_______________________________________) Area ID# [ ]

Household ID# [ ] Survey ID# [ ]

*Water Samples Collected:*

Source: 1) Normal Water: Yes No

Source: 2) Drinking Water: Yes No

**Part A: Personal General Information**

| **1** | Name of informant |  | **CODES**  Self-1  Spouse of head-2  Married child-3  Spouse of married child- 4  Unmarried child-5  Grandchild-6  Father/mother-7  Brother/sister-8  Others-9 |
| --- | --- | --- | --- |
| 2 | Informant’s relation to household head (pick code from last column)* |  |  |
| 3 | Age of informant (in years) | **Years:** |  |
| 4 | What is the highest level of education you have completed? (in years) | **Years:** |  |
| 5 | How many people, including yourself, live in your immediate household?( # of persons) **(A household is defined to comprise all usual residents, where they sleep and share common facilities)** | **Males :**  **Females :**  **Children :** |  |
| 6A | What is the total monthly cash expenditure of the household? (in Pak Rupees) | **RS:** |  |
| 6B | What is the value of home grown products used in the household (Agriculture, poultry, dairy products etc.)? (in Pak Rupees) | **RS:** |  |

***Codes***

**(THESE CODES ARE NOT TO BE TOLD TO THE RESPONDENT. CODING TO BE DONE ONLY DURING DATA ENTRY FOR THIS SECTION)**

***Q. 1.*** ***Q. 2***.

Infection – 1 Drinking water – 1

Drinking dirty water – 2 Food – 2

Eating dirty/impure food – 3 Hand – 3

Eating with dirty hands – 4 Others (specify) – 4

Others (specify) –5 Don’t know – 111

Don’t know - 111

***Q. 3***. ***Q. 4***.

Purify water – 1 Provide clean water – 1

Cook food – 2 Provide sanitation to all – 2

Wash hands before eating – 3 Collect garbage regularly – 3

Keep water and food clean – 4 Close open drains – 4

Others (specify) – 5 Public awareness & education –5

Don’t know – 111 Others – 6

***Q. 5.***

School – 1

Family – 2

Neighbors – 3

Newspaper, TV, Radio – 4

Books – 5

Others – 6

**PART B: Block 1: Awareness Module:**

*Questions 1,2, 3 and 4 are open-ended. Note down all possible answers from the respondent.*

1. What do you think causes diarrhea? (If no response, ask again)

2. If Answer to “1” includes “infection”, how is infection transmitted?

3. What do you think you should do to prevent diarrhea in your household? (If no reply, ask again: Is there nothing you can do?)

1. What do you think the municipality/government (local) can do to prevent diarrhea?

5. From whom did you acquire this information?

**Block- 2:**

**2.A Diarrhea and Filtration Related Questions:**

| **Questions** | **Responses** |
| --- | --- |
| 1) Have you ever heard of a special product called (local term or ORS) you can get for the treatment of diarrhea? If never heard of ORS, show govt. and commercial ORS packet and ask: Have you ever seen a packet like this one before? | Yes without showing the packet  Yes after showing the packet  No |
| 2) When a person has diarrhea, should he/she be given less water to drink, about the same amount, or more than usual? | Less  Same  More  DK |
| 3) Do you treat the patient’s drinking water?, if “No” skip to Block-B | Yes No |
| 4) If Q. (3) is “Yes”, how? | Straining  Boiling  Tablets/Drops  Non-electric Filter    Electric filter |

**2.B. Water Supply Related Questions:**

| Source of Drinking Water | Amount of consumption  All/ Almost all – 1  About half – 2  Little – 3 | Distance to Source  (Code) | Do you have to pay for water?  Yes – 1  No – 0  If “No”, skip to Q.5 | How much do you pay  (in PKR) per month? | Perception of Water Quality  Good - 1  Bad – 0  Don’t know – 999 | Combining all the sources, do you get sufficient water supply in your household?  Yes – 1  No - 0 |
| --- | --- | --- | --- | --- | --- | --- |
|  | **1** | **2** | **3** | **4** | **5** | **6** |
| Tap |  |  |  |  |  |  |
| Deep Tubewell |  |  |  |  |  |  |
| Shallow pump |  |  |  |  |  |  |
| Tanker |  |  |  |  |  |  |
| Bottled water |  |  |  |  |  |  |
| Other(Specify) |  |  |  |  |  |  |

Codes:

Q.(2): **Distance to Source:**

Within dwelling – 1 Outside premises between 0.2 to 0.5 km – 4,

Outside dwelling but within premises – 2, Outside premises between 0.5 to 1 km – 5,

Outside premises < 0.2 km – 3 Outside premises more than 1km – 6.

**2.B Water Supply Related Questions: (Continued)**

| Source of Drinking Water | Treatment of water (No – 0, Yes: all drinking water – 1, Yes: some drinking water – 2) | | | | |
| --- | --- | --- | --- | --- | --- |
|  | Straining | Tablets/Drops | Non-electric Filter | Electric Filters | Boiling |
|  | **7** | **8** | **9** | **10** | **11** |
| Tap |  |  |  |  |  |
| Deep Tubewell |  |  |  |  |  |
| Shallow pump |  |  |  |  |  |
| Tanker |  |  |  |  |  |
| Bottled water |  |  |  |  |  |
| Other (Specify) |  |  |  |  |  |
| Cost of filtration |  |  |  |  |  |

| 12A.If answer to Q. “11” is “1”, then, indicate the fuel used to boil the water? | Kerosene Wood LPG  Electric heater Others |
| --- | --- |
| 12B. Amount of time (in minutes) water is boiled for? |  |
| 13. Material of the main container in which water is stored? | Earthen Plastic Other non-metal Iron Copper  Stainless Steel Brass Other Metal No storage |
| 14. How is water taken out from the main container? | Tap Poured Out Vessel without handle  Vessel with handle dipped in to take out water |
| 15. What do like least about your water supply? | Bad taste / odor Cloudy Other Nothing |

**Block 3. Housing Characteristics:**

| **(1)** Nature of House: | Kutcha Semi Pucca Pucca. |
| --- | --- |
| **(2)** Surroundings of the House | Clean Average Dirty |
| **(3)** Number of Rooms | One Two Three to Five More than Five |
| **(4)** Electricity in the House (If “No”, skip to Q. 6) | Yes No |
| **(5)** In the week before survey, average no of hours without power per day. | Hours: |
| **(6)** Separate kitchen in House: | Yes No |
| **(7)** Type of Cooking: | - Ordinary Chulha/ Any other smoke emitting stove   Smokeless Chulha Kerosene LPG/ Other Non- Smoking Stoves |
| **(8)** Chimney/smoke outlet in the cooking place: | Yes No |
| **(9)** If housing owned / Rented? | Owned Rented Other |
| **(10)** Type of latrine: | None Service Latrine Flush Latrine Other (specify) |
| **(11)** Wastes discharging into: | Sewer System Septic Tank Pit  Drain or River Don’t Know |
| **(12)** Distance from the latrine used: | Within dwelling Outside dwelling but within premises  Outside premises but at a distance < ½ km Beyond ½ km |
| **(13)** Mode of collection of garbage from house: | By local authority By private arrangements among residents  By household members Others |
| **(14)** Frequency of garbage clearance: | Daily Not daily but at least once a week  Not even once a week DK |
| **(15)** Are you concerned about the problem of flies/ mosquitoes/ foul odor in your area? | Yes No There is no such problem |
| **16)** Are you willing to contribute towards improving sanitation in your neighborhood village/ town? | Yes No There is no such problem |

**Block 4. Household Members’ Health Treatment:**

**Codes:**

**Q. (2): Treatment Place*:*** **Q. (5): Usual Activity**

Public – 1 Public service – 1

NGO – 2 Private service – 2

Private doctor (MBBS) – 3 Own business – 3

Private doctor (not MBBS) – 4 Others – 4.

Private non-allopathic – 5

Nowhere – 6

**Q. (3): Treatment Mode*:***

ORS – 1 Pill or syrup – 2

Injection – 3 Intravenous (I.V./ Drip/ Bottle) – 4

Homemade sugar-salt-water solution – 5 Herbal Medicine – 6

Others (Specify) – 7 Nothing *– 8*

**Household Members’ Health Treatment:**

| Serial No. | Name of Member | Has (Name) had diarrhea  in the last one month?  Yes – 1  No – 0  DK – 3  If “No” or “DK”,  skip to Q. 5 | Where did you seek treatment or advice?  (Code)  (Record all that are mentioned) | What treatments?  (Code) | If the individual was absent from work for the first episode of diarrhea, the duration of absence in days | Usual activity  (code) |
| --- | --- | --- | --- | --- | --- | --- |
|  |  | **(1)** | **(2)** | **(3)** | **(4)** | **(5)** |
| 1 |  |  |  |  |  |  |
| 2 |  |  |  |  |  |  |
| 3 |  |  |  |  |  |  |
| 4 |  |  |  |  |  |  |
| 5 |  |  |  |  |  |  |
| 6 |  |  |  |  |  |  |
| 7 |  |  |  |  |  |  |
| 8 |  |  |  |  |  |  |
| 9 |  |  |  |  |  |  |
| 10 |  |  |  |  |  |  |

# Appendix B: Handout given to households in Full treatment, Wave 2

**COMSATS INSTITUTE OF INFORMATION TECHNOLOGY, LAHORE**

Dear Madam/Sir,

We took drinking water samples to test the water quality in your home during the last survey. Thank you for your cooperation. We are returning the samples.

The color of the water samples will be yellow or purple. If the color is purple it is most likely that your water does not contain bacteria. If the color is yellow, then it is likely that the water is contaminated with germs that may cause diseases like Typhoid, dysentery, hepatitis, cholera and giardiasis. **But this simple test cannot confirm that the water is contaminated**. You may wish to take the following preventive measures:

1. Water can get contaminated quite easily within the home, so keep your drinking water storage containers clean and covered at all times. If your water storage container does not have a tap to take out the water, use a clean utensil with a long handle to take out the water. **Never dip your hands into the water storage container.**

2. Consider using a home water purification method that fits your household’s budget. The different methods available and their average prices in Lahore and Sheikhupura are:

| Method | Equipment Cost (Rs.) | Operating cost (Rs.) | Features |
| --- | --- | --- | --- |
| Straining with clean cotton cloth folded 8 or more times |  |  | Limited protection against germs |
| Disinfecting tablets/drops |  |  | Kills nearly all germs |
| Non-electric filters |  |  | Removes some germs depending on the fineness of the filter |
| Electric filters |  |  | Kills all germs with UV rays if properly maintained |
| Boiling |  |  | Kills all germs |
| Drink bottled water |  |  | Manufacturer’s responsibility to ensure germ-free water. |
| Reverse Osmosis (RO) |  |  | Removes all germs if properly maintained. |

3. If using purification methods like tablets, non-electric or electric filters, follow the manufacturer’s operating and maintenance instructions carefully as well as the water-handling precautions mentioned above.

Dr. [[hidden for blind review]]]

…

,

COMSATS Institute of Information Technology,

Lahore, Pakistan.


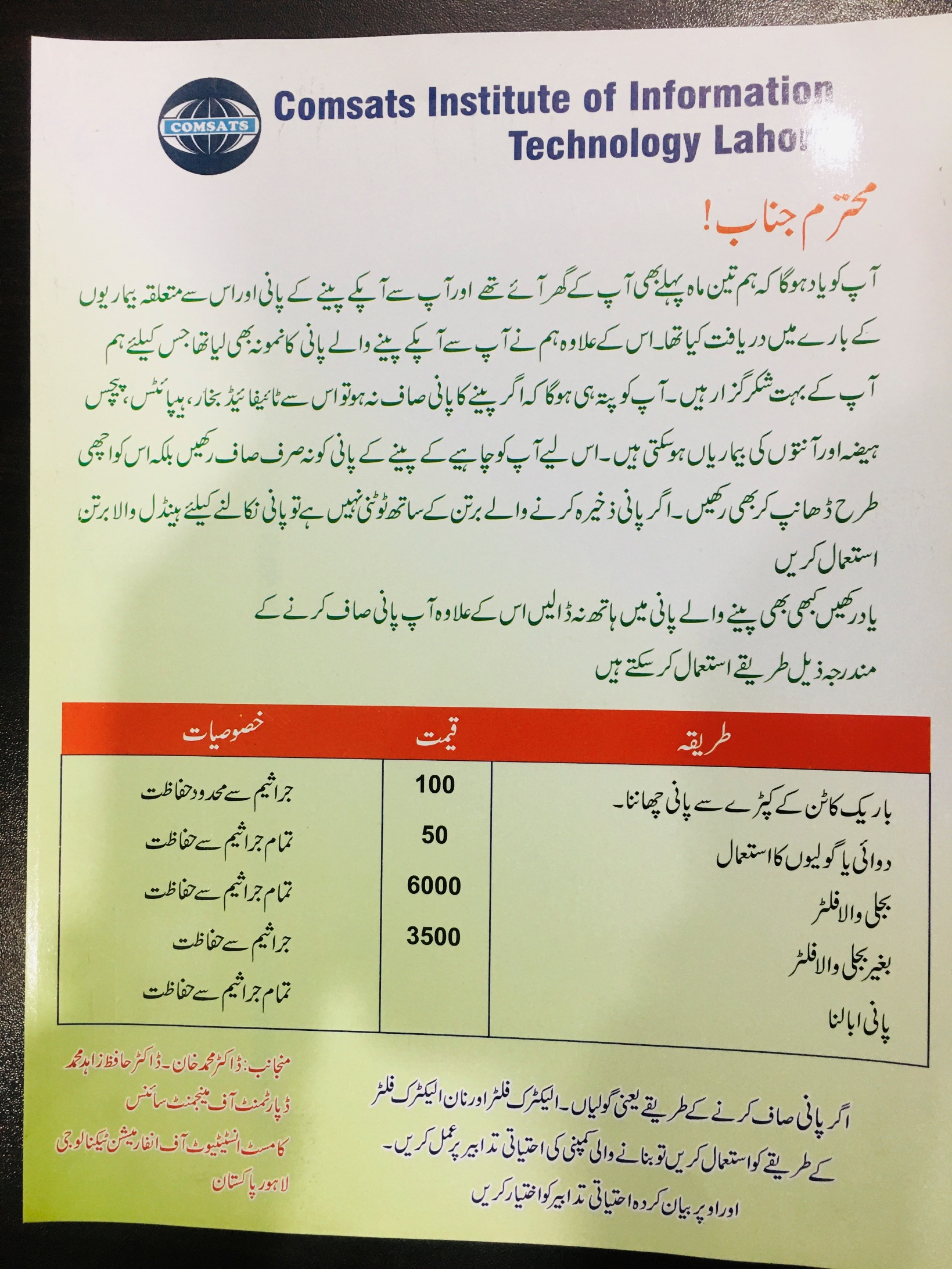


# Appendix C: Implementation of water purification measures questionnaire, Wave 3

Dear Madam/Sir, Date of third visit:

We had visited your household in….Months…… and again revisited your household in ….Months…… to inform you about your water test results as well as to provide you some information on how you can safeguard yourself and your family from health hazards arising from drinking contaminated water (last part to be told to the treatment households only). This is the final visit of our study and we would like to ask you a few follow-up questions that will not take more than 5 minutes of your time. We really appreciate your patience and co-operation during the course of our survey. Thank you

1. In our first visit, during the household survey, you had mentioned that you were using _______ purification method/ not using any purification method. Since that visit did you make any changes in the water purification methods you use? Yes No
2. If question to (1) is “no” go to question 4. If yes, what kind of changes did you make?

Adopted new purification method _______________________

Do not use any purification method any more

Others (specify)

1. If answer to question (2) is “adopted new purification method” specify:

Method: Brand name: Fixed cost of the equipment:

1. (Ask only of those households who were previously using either a non-electronic filter or an electronic filter and have not changed their water purification method)

(a) Since our first visit, have you gotten the candles changed (for non-electronic filters) and/or the carbon changed (for electronic filters)? Yes No

1. Finally we would like to ask you whether you are planning to make some changes in the future to improve the quality of your drinking water? Yes No
2. If answer is “yes” to the above question, what kind of changes do you anticipate making?

When do you think you will make this change?

How much do you think you will be able to spend on such changes?

# Appendix D: Photos and instructions of the E.coli test kits

Please note that during the intervention the test was branded HydroCheck, while the name changed after to SinoW. We provide photos of the test, instructions and branding below to understand the potential ease of use of the test by households.


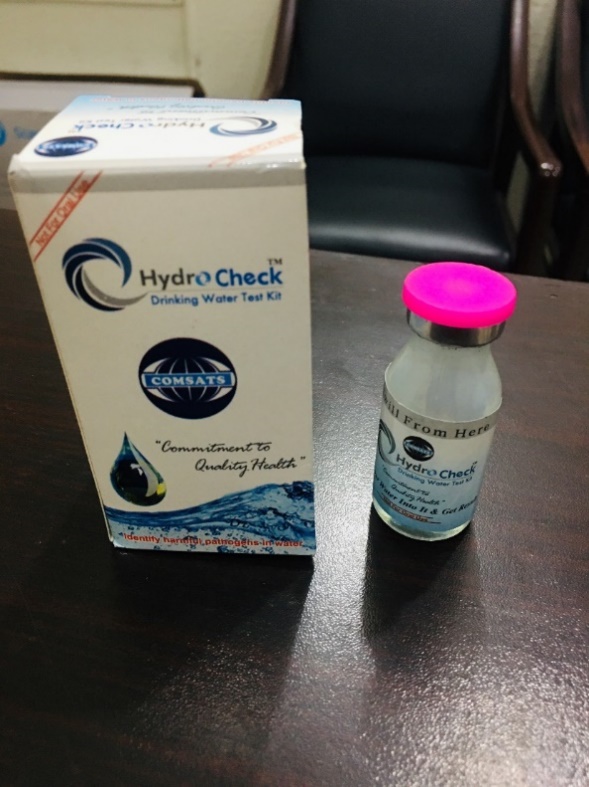


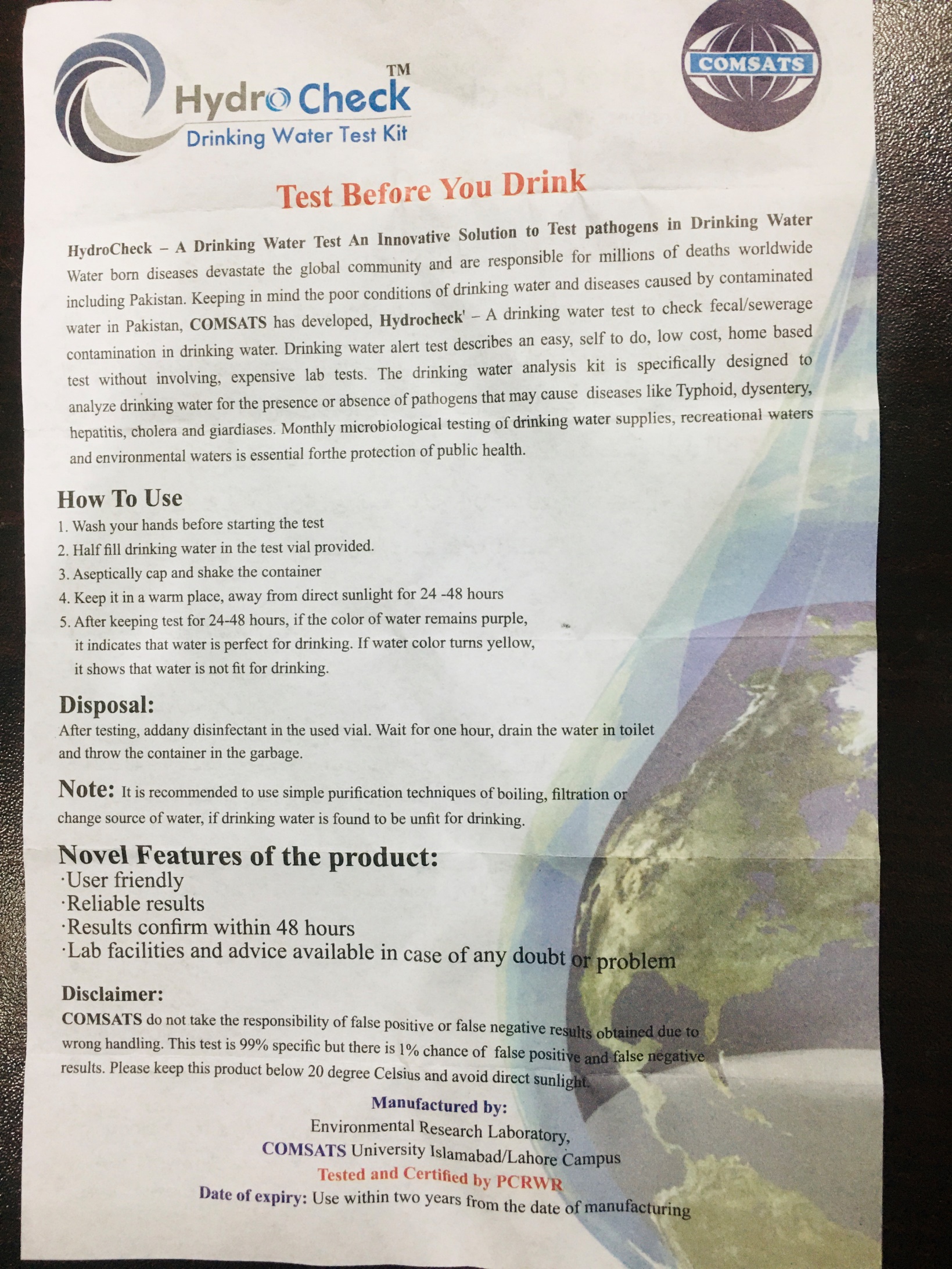

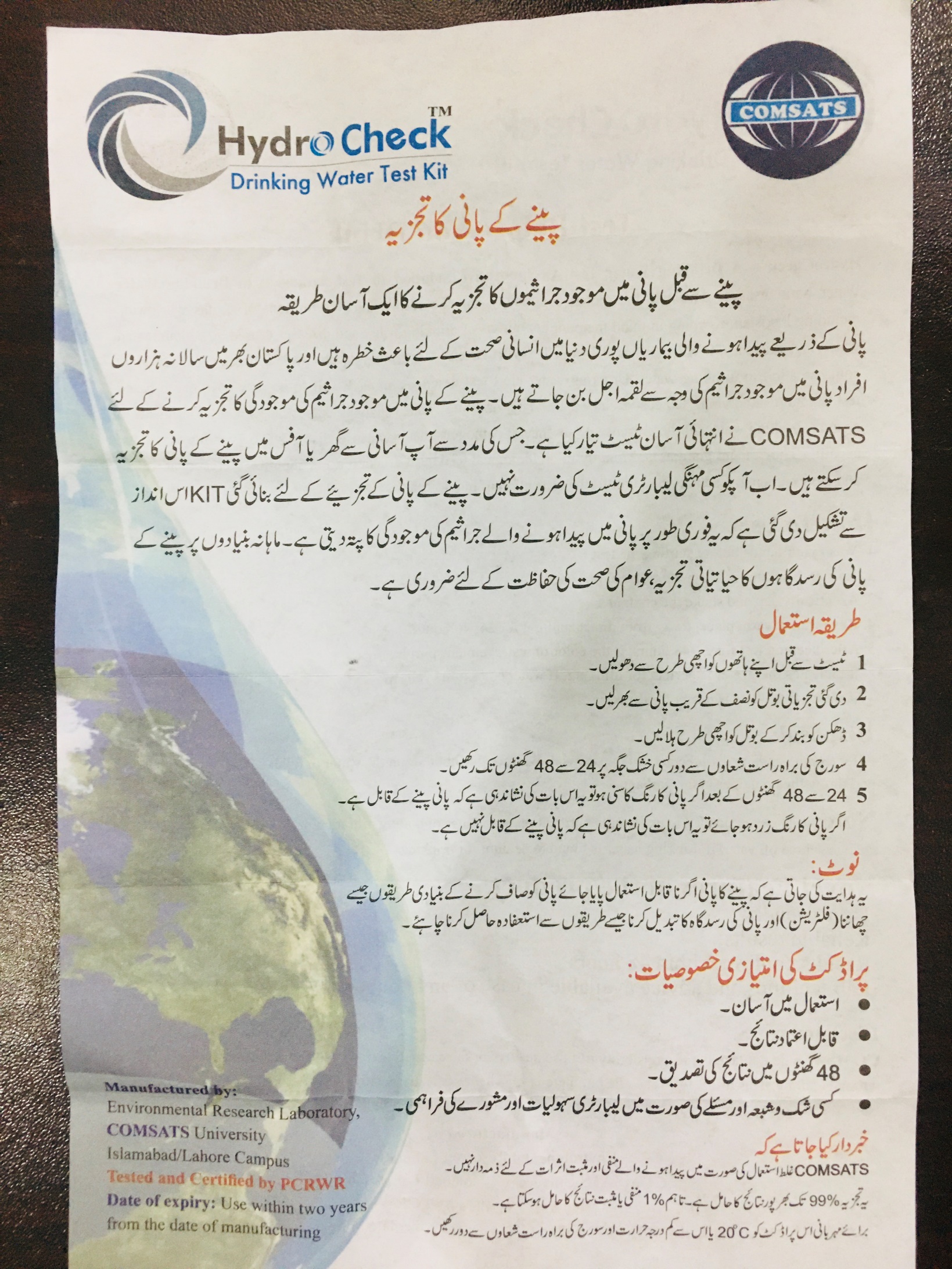


**
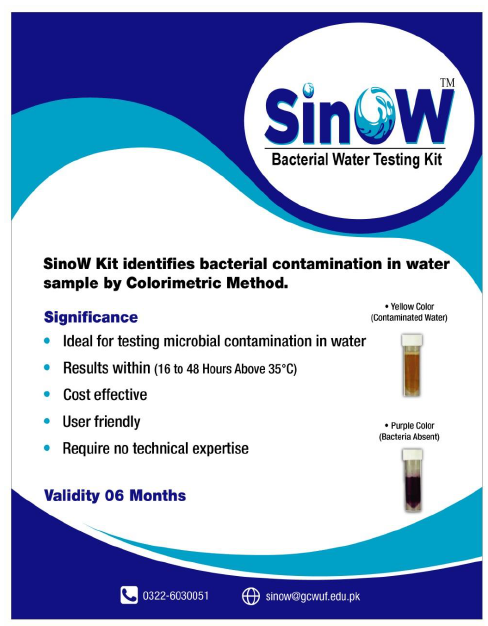

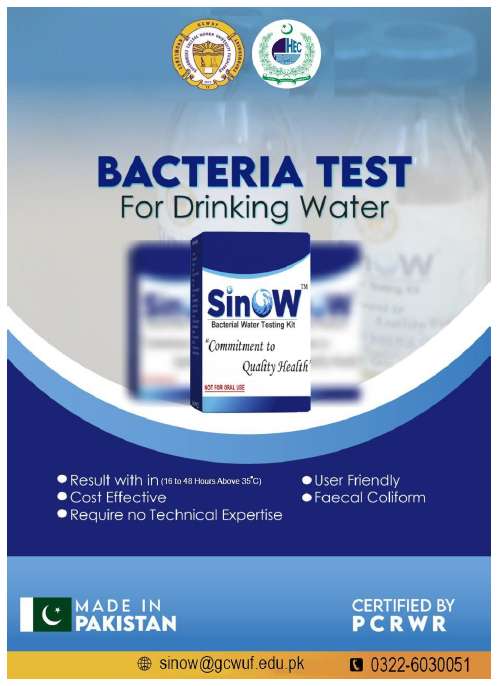

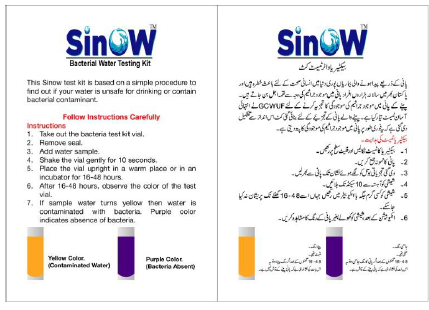
**

**
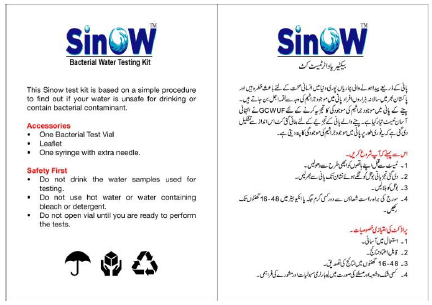
**

# Appendix E: Supplementary figures and tables

Table E1: Balance tables of observable characteristics across treatment arms in villages that

had both control and treatment conditions

|  |  |  |  | p-values for test of equality | | |
| --- | --- | --- | --- | --- | --- | --- |
|  | No treatment  (control group) | Partial Treatment | Full Treatment | *NT=PT* | *NT=FT* | *PT=FT* |
| *Socioeconomic characteristics* |  |  |  |  |  |  |
| Education of the household head (years) | 4.13 (4.38) | 5.08  (4.80) | 5.07  (4.79) | *0.50* | *0.50* | *0.89* |
| Age of the household head (years) | 48.97 (12.42) | 47.33  (12.03) | 48.52  (11.57) | *0.96* | *0.97* | *0.94* |
| People in the household | 7.58 (3.56) | 7.73  (3.20) | 7.99  (3.77) | *0.99* | *0.74* | *0.70* |
| Monthly household expenditure (in Pak Rupees) | 31,256 (17,259) | 30,239  (14,557) | 29,186  (16,683) | *0.99* | *0.99* | *0.99* |
| *House characteristics* |  |  |  |  |  |  |
| Pucca (%) | 81 | 82 | 81 | *0.97* | *0.99* | *0.88* |
| Ownership (%) | 94 | 94 | 87 | *0.95* | *0.57* | *0.60* |
| Separate Kitchen (%) | 60 | 64 | 63 | *0.98* | *0.98* | *0.97* |
| House surroundings rated dirty by the assistant (%) | 36 | 34 | 38 | *0.99* | *0.99* | *0.92* |
| *Water and sanitation* |  |  |  |  |  |  |
| Treat Drinking Water (%) | *7* | 6 | 10 | *0.92* | *0.95* | *0.28* |
| Plastic water storage (%) | 90 | 90 | 93 | *0.94* | *0.98* | *0.98* |
| Water accessed from tap (%) | 48 | 47 | 44 | *0.95* | *0.98* | *0.98* |
| Toilet discharges to a River or Drain (%) | 27 | 22 | 25 | *0.79* | *0.98* | *0.14* |
| Family Member suffered from diarrhea in the last month (%) | 30 | 29 | 31 | *0.99* | *0.72* | *0.12* |
| *Health Knowledge* |  |  |  |  |  |  |
| Diarrhea patients should use more water (%) | 49 | 57 | 55 | *0.87* | *0.97* | *0.99* |
| Patients’ water should be treated/filtered (%) | 69 | 68 | 64 | *0.99* | *0.97* | *0.99* |
| N | 144 | 326 | 242 |  |  |  |
| *Note: Standard deviations in parentheses. NT stands for No treatment (control group) PT/FT stands for Partial/Full treatment. p-values are corrected for multiple hypotheses testing procedure introduced in List et al. (2023).* | | | | | | |

Table E2: Predicting change in the implementation of water purification measures, post-intervention.

| **Probit Regressions** | All Villages | | Villages with Treatments  and Control | | |
| --- | --- | --- | --- | --- | --- |
|  | (1) | (2) | (3) | (4) | |
| Partial treatment | 0.393*** | 0.391*** | 0.396*** | 0.385*** |  |
| (water test) | (0.031) | (0.028) | (0.036) | (0.031) |  |
| Full treatment | 0.476*** | 0.508*** | 0.442*** | 0.462*** | |
| (water test + information) | (0.039) | (0.041) | (0.032) | (0.035) | |
|  |  |  |  |  | |
| *Chow test p-value PT=FT* | 0.108 | 0.017 | 0.335 | 0.094 | |
| Controls | No | Yes | No | Yes | |
| Observations | 1009 | 1002 | 712 | 705 | |
| Pseudo R^2^ | 0.202 | 0.259 | 0.128 | 0.195 | |

*Note: Marginal effects are reported. Villages with Treatments and Control are those that had households assigned to either control or treatment within a single village. No Treatment (Control condition) is the omitted category for treatment comparisons. Partial treatment (PT: water contamination results = 1) and Full treatment (FT: water contamination results + information provided = 1) are included as explanatory variables. Standard errors clustered at village level. Controls include all the variables described in Table E1 and included in Table 2. * p<0.10, ** p<0.05, *** p<0.01*


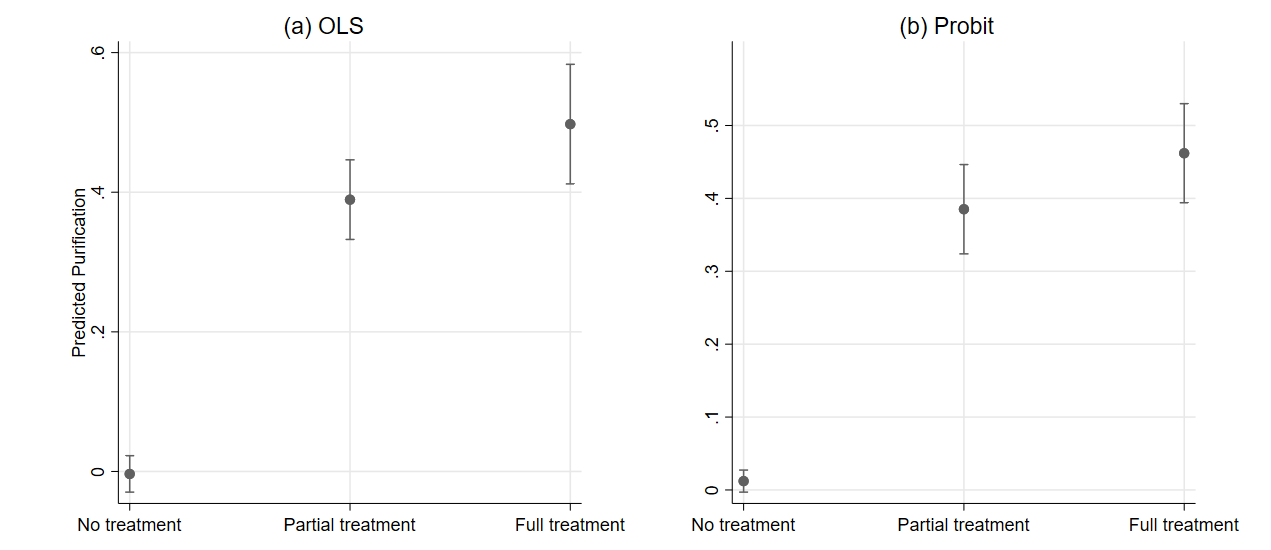


**Figure E1:** Marginal effects of each treatment (predicted likelihood of implementing water purification measures)

*Note: Error bars are 95% confidence intervals.*

**Figure E2:** Heterogeneity Analysis. Proportion of households implementing water purification measures in each treatment by subgroup (raw data)

*Note: Error bars are 95% confidence intervals.*

| **Table E3: Propensity Score Matching to Estimate Average Treatment Effects on the Implementation of Purification Measures** | | | |
| --- | --- | --- | --- |
| Matching on: | Partial vs No Treatment | Full vs No Treatment | Full vs Partial Treatment |
| Socioeconomic Characteristics | 0.369***  (0.029) | 0.455***  (0.032) | 0.072*  (0.044) |
| No of matched pairs | 4 | 4 | 3 |
| House Characteristics | 0.380***  (0.027) | 0.469***  (0.031) | 0.098***  (0.038) |
| No of matched pairs | 130 | 109 | 130 |
| Health Knowledge and Sanitation Characteristics | 0.406***  (0.031) | 0.468***  (0.037) | 0.103***  (0.039) |
| No of matched pairs | 48 | 33 | 48 |
| *Note: The reported coefficients are Average Treatment Effect with robust standard errors in parentheses.* | | | |

Table E4: Heterogenous effects of the treatments (full sample)

|  | (1) | (2) | (3) | (4) |  |
| --- | --- | --- | --- | --- | --- |
| Partial Treatment | 0.399*** | 0.335*** | 0.343*** | 0.412*** |  |
|  | (0.044) | (0.055) | (0.035) | (0.046) |  |
| Full Treatment | 0.493*** | 0.492*** | 0.500*** | 0.424*** |  |
|  | (0.057) | (0.060) | (0.042) | (0.047) |  |
| High_Education | -0.046 |  |  |  |  |
|  | (0.044) |  |  |  |  |
| Partial treatment × High_Edu | -0.012 |  |  |  |  |
|  | (0.053) |  |  |  |  |
| Full Treatment × High_Edu | 0.019 |  |  |  |  |
|  | (0.061) |  |  |  |  |
| High_Expenditure |  | -0.060 |  |  |  |
|  |  | (0.036) |  |  |  |
| Partial treatment × High_Expenditure |  | 0.113** |  |  |  |
|  |  | (0.051) |  |  |  |
| Full Treatment × High_Expenditure |  | 0.017 |  |  |  |
|  |  | (0.057) |  |  |  |
| Water Tap |  |  | 0.005 |  |  |
|  |  |  | (0.021) |  |  |
| Partial treatment × Water_tap |  |  | 0.066 |  |  |
|  |  |  | (0.045) |  |  |
| Full Treatment × Water_tap |  |  | -0.007 |  |  |
|  |  |  | (0.029) |  |  |
| No Recent Diarrhea |  |  |  | 0.017 |  |
|  |  |  |  | (0.029) |  |
| Partial treatment × No Recent Diarrhea |  |  |  | -0.056 |  |
|  |  |  |  | (0.057) |  |
| Full Treatment × No Recent Diarrhea |  |  |  | 0.096 |  |
|  |  |  |  | (0.077) |  |
| Constant | -0.177 | -0.168 | -0.213 | -0.172 |  |
|  | (0.171) | (0.173) | (0.210) | (0.173) |  |
| Observations | 1002 | 1002 | 934 | 936 |  |
| Controls | Yes | Yes | Yes | Yes |  |
| R^2^ | 0.242 | 0.244 | 0.233 | 0.233 |  |
| *Note: Standard errors from OLS regressions clustered at village level are in parentheses. No Treatment (Control condition) is the omitted category for treatment comparisons. Heterogenous treatment effects. Same set of controls are used in all regressions as in Table 1 in the main text. * p<0.10, ** p<0.05, *** p<0.01* | | | | | |
|  | | | | | |

Table E5: Heterogenous effects of the treatments (villages with treatments and control)

|  | (1) | (2) | (3) | (4) |
| --- | --- | --- | --- | --- |
| Partial Treatment | 0.377*** | 0.299*** | 0.345*** | 0.380*** |
|  | (0.045) | (0.051) | (0.039) | (0.048) |
| Full Treatment | 0.428*** | 0.416*** | 0.469*** | 0.412*** |
|  | (0.056) | (0.055) | (0.046) | (0.051) |
| High_Education | -0.103 |  |  |  |
|  | (0.062) |  |  |  |
| Partial treatment × High_Edu | 0.014 |  |  |  |
|  | (0.059) |  |  |  |
| Full Treatment × High_Edu | 0.062 |  |  |  |
|  | (0.072) |  |  |  |
| High_Expenditure |  | -0.128** |  |  |
|  |  | (0.048) |  |  |
| Partial treatment × High_Expenditure |  | 0.162*** |  |  |
|  |  | (0.041) |  |  |
| Full Treatment × High_Expenditure |  | 0.078 |  |  |
|  |  | (0.069) |  |  |
| Water Tap |  |  | 0.009 |  |
|  |  |  | (0.033) |  |
| Partial treatment × Water_tap |  |  | 0.057 |  |
|  |  |  | (0.048) |  |
| Full Treatment × Water_tap |  |  | -0.031 |  |
|  |  |  | (0.032) |  |
| No Recent Diarrhea |  |  |  | -0.023 |
|  |  |  |  | (0.036) |
| Partial treatment × No Recent Diarrhea |  |  |  | -0.008 |
|  |  |  |  | (0.066) |
| Full Treatment × No Recent Diarrhea |  |  |  | 0.063 |
|  |  |  |  | (0.062) |
| Constant | -0.100 | -0.084 | -0.112 | -0.088 |
|  | (0.217) | (0.218) | (0.279) | (0.228) |
| Observations | 705 | 705 | 677 | 679 |
| Controls | Yes | Yes | Yes | Yes |
| R^2^ | 0.191 | 0.194 | 0.189 | 0.188 |
| *Note: Standard errors from OLS regressions clustered at village level are in parentheses. Partial treatment is the benchmark group. Heterogenous treatment effects. Same set of controls are used in all regressions as in Table 1 in the main text. * p<0.10, ** p<0.05, *** p<0.01* | | | | |
|  | | | | |
